# Propranolol, a β-adrenoceptor antagonist, worsens liver injury in a model of non-alcoholic steatohepatitis

**DOI:** 10.1016/j.bbrc.2013.07.005

**Published:** 2013-08-09

**Authors:** Chad McKee, Junpei Soeda, Esra Asilmaz, Barbara Sigalla, Maelle Morgan, Nicoletta Sinelli, Tania Roskams, Jude A. Oben

**Affiliations:** a Institute for Liver and Digestive Health, University College London, Royal Free Campus, London NW3 2PF, United Kingdom; b Department of Morphology and Molecular Pathology, University of Leuven, Belgium; c Department of Gastroenterology and Hepatology, Guy’s & St. Thomas’ Hospital, London SE1 7EH, United Kingdom

**Keywords:** PRL, propranolol, PRZ, prazosin, NASH, non-alcoholic steatohepatitis, NAFLD, non-alcoholic fatty liver disease, OC, oval cell, HPC, hepatic progenitor cell, Non-alcoholic fatty liver disease, Propranolol, Sympathetic nervous system, Hepatocytes, Apoptosis

## Abstract

Prazosin an α1-adrenoceptor (AR) antagonist has been shown to reduce liver injury in a mouse model of non-alcoholic steatohepatitis (NASH) and is suggested as a potential treatment of NASH especially given its concomitant anti-fibrotic properties. The effect however, of β-AR blockade in non-cirrhotic NASH is unknown and is as such investigated here. In the presence of the β-blocker propranolol (PRL), mice fed normal chow or a half methionine and choline deficient diet, supplemented with ethionine (HMCDE), to induce NASH, showed significantly enhanced liver injury, as evidenced by higher hepatic necrosis scores and elevated serum aminotransferases (ALT). Mechanistically, we showed that murine hepatocytes express α and β adrenoceptors; that PRL directly induces hepatocyte injury and death as evidenced by increased release of lactate dehydrogenase, FASL and TNF-α from hepatocytes in the presence of PRL; and that PRL activated the apoptotic pathway in primary hepatocyte cultures, as indicated by upregulation of Fas receptor and caspase-8 proteins. The β-AR antagonist PRL therefore appears to enhance liver injury through induction of hepatocyte death via the death pathway. Further studies are now required to extrapolate these findings to humans but meanwhile, β-AR antagonists should be avoided or used with caution in patients with non-cirrhotic NASH as they may worsen liver injury.

## Highlights

• β-Blocker propranolol worsens liver injury in model of non-alcoholic steatohepatitis.
• Mechanism of hepatic injury is via activation of apoptotic pathway in hepatocytes.
• β-Blockers should be avoided or used with extreme caution in patients with NASH.

## 1 Introduction

The prevalence of non-alcoholic fatty liver disease (NAFLD) is rising worldwide in parallel with increasing rates of obesity [1], [2]. At present, about 23–34% of the United States population have NAFLD: with the population prevalence of the more severe stage – NASH, estimated at about 2.5% [3]. In the United Kingdom, adult obesity rates have trebled since 1980 [4] and are predicted to reach 50% by 2050 [5]. The prevalence, moreover, of NAFLD in obese or diabetic individuals is higher than in the general population and is estimated at 70–90% [6]. NAFLD defines a spectrum of liver diseases from fatty liver (steatosis), through fatty liver with inflammation (NASH), to cirrhosis and primary liver cancer [2]. Despite its demographic importance, proven treatments are currently not available.

The sympathetic nervous system (SNS) appears to be a viable therapeutic target in NAFLD, as α1-adrenoceptor antagonism with prazosin (PRZ) has been shown to reduce liver injury, in a model of NASH [7]. This suggests that the homeostatic effect of SNS signaling on liver repair is inhibitory such that adrenoceptor antagonism enhanced repair. Furthermore, hepatic fibrogenic stellate cells express functional adrenoceptors [8], [9], [10] and *Dbh*^−/−^ mice lacking catecholamine SNS neurotransmitters through deletion of the catecholamine synthesizing enzyme dopamine β-hydroxylase as well as ob/ob mice, which are deficient in leptin with reduced SNS neurotransmitter tone, have impaired fibrogenic responses [8]. These altogether suggest that adrenoceptor antagonists offer utility as novel, pro-regenerative, anti-fibrotic, treatments for NAFLD. Although there is a large body of liver experience with β-AR antagonists, such as propranolol (PRL), which are used for treatment of portal hypertension [11], [12], [13], [14], their effect in non-cirrhotic liver, in NAFLD is not known.

To address this, we used a mouse model of NASH, where we showed that PRL, unlike prazosin, increased biochemical and histological evidence of liver injury. We next investigated the mechanisms through which PRL induced liver injury in vitro and showed that PRL induces liver injury via activation of apoptotic pathways.

## 2 Materials and methods

### 2.1 Animals

Male, lean C57BL/6 mice, and ob/ob mice, 10–18 weeks old were from Jackson Laboratory (Bar Harbor, ME). Animals had unrestricted access to diets and water. All animal experiments satisfied National and Institutional regulatory guidelines.

### 2.2 Diets and drugs

Control methionine choline diet (CMCD) was purchased from ICN (Aurora, OH). The injurious diet, also from ICN, contained half the control amounts of choline and methionine and was administered with 0.15% ethionine (Sigma, St. Louis, MO) in drinking water, previously shown to enhance oxidative injury to the liver and cause hepatic accumulation of OC [7], [15]. The combination treatment is hereafter referred to as half methionine choline-deficient diet plus ethionine (HMCDE).

### 2.3 In vivo experiments

For the in vivo PRL experiments, lean normal mice were divided into 4 groups (10–12 mice/group) and treated as follows: Normal chow, NC (control diet); NC plus intra-peritoneal (IP) administration of PRL (Sigma, St. Louis, MO), at 4 mg/kg/day; HMCDE diet alone; and HMCDE plus IP administration of PRL, all for 4 weeks. This protocol is essentially as used previously [7].

All mice were weighed at the beginning of the feeding period and weekly thereafter until sacrifice. At sacrifice, livers were fixed in buffered formalin or optimal cutting temperature (OCT) fixative (Sakura, Torrance, CA) and processed for histology. Alternatively, liver samples were snap frozen in liquid nitrogen and stored at −80 °C for further analysis.

### 2.4 Histology

Sections of liver were prepared for histology and immunochemistry as previously described [16], [17]. Hematoxylin–eosin-stained sections were examined by an experienced liver pathologist blinded to treatment groups. Hepatocellular fat accumulation was scored in the following manner: no fat = 0, focal fat accumulation in <0.1% of the hepatocytes = 1, fat in 1–30% of the hepatocytes = 1+, fat in 31–60% of the hepatocytes = 2+, and fat in 61–100% of the hepatocytes 3+. To evaluate the amount of hepatocyte necrosis, the number of necrotic hepatocytes was counted in 10 randomly selected fields with a 20× lens.

### 2.5 Culture media

All primary cells isolated were grown in either control medium (DMEM), supplemented with fetal bovine serum (FBS), or a methionine and choline deficient (MCD) medium, as an in vitro equivalent of the in vivo administered HMCDE. The MCD medium has previously been shown to induce steatosis and injury in the hepatocyte cell line AML-12 [18].

### 2.6 Isolation and culture of mouse primary hepatocytes

Mouse primary hepatocytes were obtained each time from the livers of 3 sacrificed mice. The livers were gently crushed in the presence of collagenase type IV (10 μM, Sigma) and DNase (10 μM, Sigma) in HBSS (Gibco), followed by a 20 min shaking incubation at 37 °C. The homogenate was then filtered, into fresh tubes, and centrifuged at 500*g* for 3 min. The supernatant was discarded, the pellet washed with PBS and then re-suspended in red cell lysis buffer (e-biosciences, San Diego, CA) and incubated at room temperature for 10 min, followed by centrifugation at 500*g* for 3 min. The obtained hepatocytes were re-suspended in 1 mL of DMEM prior to inoculation into 12 well plates coated with collagen type I (# 35,6700, BD Biosciences, San Jose, CA). Hepatocytes were cultured either in DMEM with/without FBS, ±PRL or in MCD with/without FBS, ±PRL.

### 2.7 Alanine Aminotransferase

Supernatants from primary hepatocyte cultures were analyzed for Alanine Aminotransferase (ALT) content by our Institutions’ Clinical Chemistry Laboratory.

### 2.8 Lactate dehydrogenase

Approximately 5 × 10^4^ primary hepatocytes were inoculated into each well of a 96 well plate in triplicate. Following appropriate treatments, each sample was homogenized in lysis buffer, incubated for 10 min (5% CO2, 90%, humidity, 37 °C) and the lysates centrifuged at 600*g*. 10 μl aliquots of supernatant were then transferred to a fresh 96 well plate, 100 μl of LDH reaction mixture (Biovision, K313-600) was added and the samples incubated for 30 min at room temperature after which optical density at 450 nm was determined and LDH concentration extrapolated from a standard curve.

### 2.9 TNF-α and FAS-ligand

TNF-α and FASL proteins were quantified with the Quantikine sandwich ELISA system (#MTA00 & #MFL00, R&D Systems, Minneapolis, MN). Briefly, supernatants from appropriately treated primary hepatocytes grown in control or MCD media were centrifuged at 1500 rpm to remove debris and transferred to 96 well plates. 50 μl of supernatant was then incubated in the presence of a proprietary conjugate and colorimetric substrate as instructed by the manufacturer. The optical density at 570 nm was then determined in an Anthos htIII spectrometer and analyte concentration extrapolated from a standard curve.

### 2.10 RT-PCR

RNA was isolated from murine hepatocytes using either Trizol reagents (Invitrogen) or RNeasy kits (Qiagen, Valencia, CA). Concentration and purity were assessed by absorbance at 260/280 nm. RT-PCR was performed with superscript one-step with platinum Taq kits (Invitrogen, Carlsbad, CA) using Ambion’s QuantumRNA Classic II 18S internal standard (Ambion, Austin, TX). Primer sequences and conditions were as reported [7].

Final PCR products were analyzed both by melting curves and agarose gel (1%) electrophoresis. The amount of transcript was calculated and expressed as the difference relative to the control gene GAPDH (2^−ΔΔCt^, where ΔCt represents the difference in threshold cycles between the target and control genes) essentially as described [7].

### 2.11 Western blotting

Proteins of the apoptotic pathways were evaluated in primary hepatocytes after treatments with PRL as described above. Cell homogenates were prepared and protein content quantified by BSA assay (Pierce, Rockford, IL) using bovine serum albumin standards. Proteins (20 μg/lane) were then resolved by polyacrylamide gel electrophoresis, transferred to nylon membranes and probed with the following primary antibodies: caspase-3, #9662; caspase-9, #9509; cytochrome c, #4272 (all supplied by Cell Signaling); caspase-8, AF1650 (R&D Systems), FAS, AF9150 (R&D Systems), with β-actin, SC-1616 (Santa Cruz Biotechnology, Santa Cruz, CA) as a control.

Horseradish peroxidase-conjugated secondary antibodies, SC-2031 (Santa Cruz Biotechnology) detected primary antibodies and antigens were demonstrated by enhanced chemiluminescence (Amersham Pharmacia Biosciences, Piscataway, NJ).

### 2.12 Statistical analysis

All values are expressed as mean ± SEM. Group means were compared by unpaired *t*-test or ANOVA using Graphpad Prism 3.03 (San Diego, CA). Significance was accepted as *p* < 0.05.

## 3 Results

### 3.1 PRL induces liver injury in NC or HMCDE fed mice

**Fig. 1:**
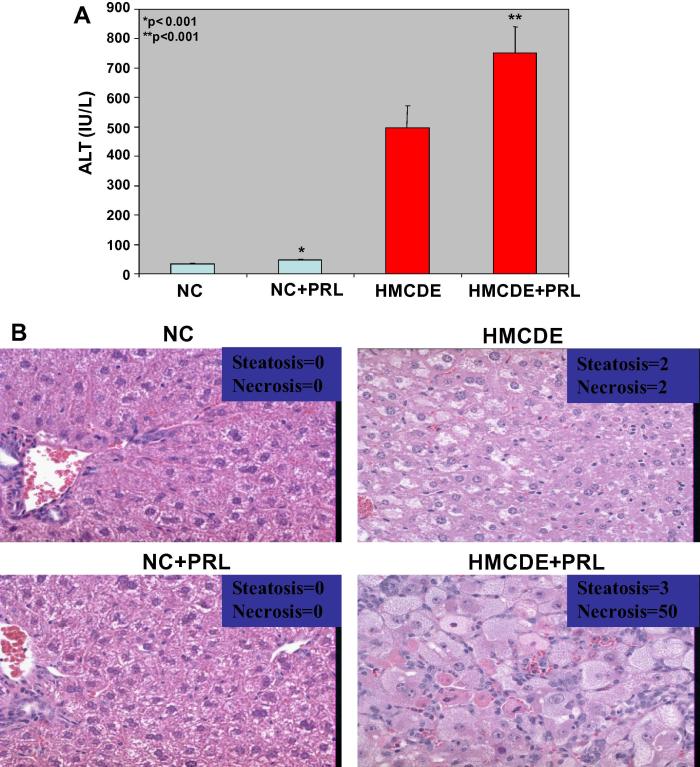
(A, B): PRL increases HPC numbers and markers of liver injury in mice fed normal chow or HMCDE as a model of NASH. Mice were fed either a control diet (normal chow, NC) or a diet containing half the control amounts of choline and methionine, supplemented with ethionine in drinking water (HMCDE), to induce steatohepatitis, hepatocyte growth arrest. After 4 weeks, liver samples were obtained, fixed in formalin and paraffin-embedded. In addition, sera were assayed for ALT as a marker of liver injury and confirmed by histological assessment for extent of necrosis. 5–8 mice per group were used in each of 2 separate experiments. The results from both experiments were similar. Parameters are graphed as mean ± SEM. OC numbers: (a) ALT levels. ^∗^*p* < 0.001 for NC versus NC+PRL, *n* = 8 per group and ^∗∗^*p* < 0.001 for HMCDE versus HMCDE+PRL, *n* = 8 per group. (b) Representative liver histology from a mouse in each of the 4 groups scored for fat and extent of necrosis, confirming biochemical evidence of PRL induction of liver injury in mice fed HMCDE.

PRZ is known to induce expansion of HPC and reduce liver injury in HMCDE fed mice [7]. To determine whether β-blockade with PRL induced a similar reduction of injury, livers of PRL treated animals were examined for extent of injury. As shown in Fig. 1A, mice fed NC diet had normal levels of ALT (about 40 IU/ml), whereas HMCDE diet induced a >10-fold increase in ALT, up to 500 IU/ml. PRL significantly increased serum ALT in both NC and HMCDE fed groups (up to 75 and 725 IU/L respectively). Histological examination of the livers in the two groups confirmed that PRL markedly enhanced liver injury (Fig. 1B).

### 3.2 Murine hepatocytes express α and β adrenoceptors

**Fig. 2:**
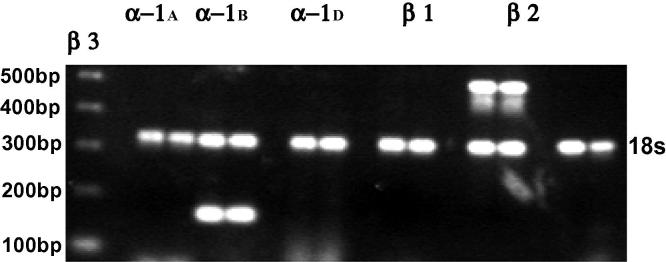
Murine hepatocytes express α and β adrenoceptors. RT-PCR of hepatocyte RNA was used to analyze expression of adrenoceptor mRNA. Results from a representative analysis are shown. The first lane shows the DNA ladder (500–200 bp). Each subsequent pair of lanes is a replicate analysis of adrenoceptor gene expression. The 18S band (324 bp) in each lane is shown as a control.

To study the differential effects of PRZ and PRL on liver injury in the context of NASH, we first assayed the presence of adrenoceptors on hepatocytes. Adrenoceptors are known to be expressed in the liver [19], [20] but whether hepatocytes express adrenoceptors and the subtypes therein is not known. Hepatocytes were shown here to express α1_B_ and β2 adrenoceptors (Fig. 2).

### 3.3 PRL directly induces hepatocyte injury

The increase in liver damage engendered by PRL suggested that PRL treatment in the context of NASH may directly induce hepatocyte injury. To confirm this possibility, mouse primary hepatocytes were cultured in control or MCD media (in order to simulate NASH in vitro) [18], in the presence or absence of PRL. Hepatocyte injury was assessed by assaying released LDH.

**Fig. 3:**
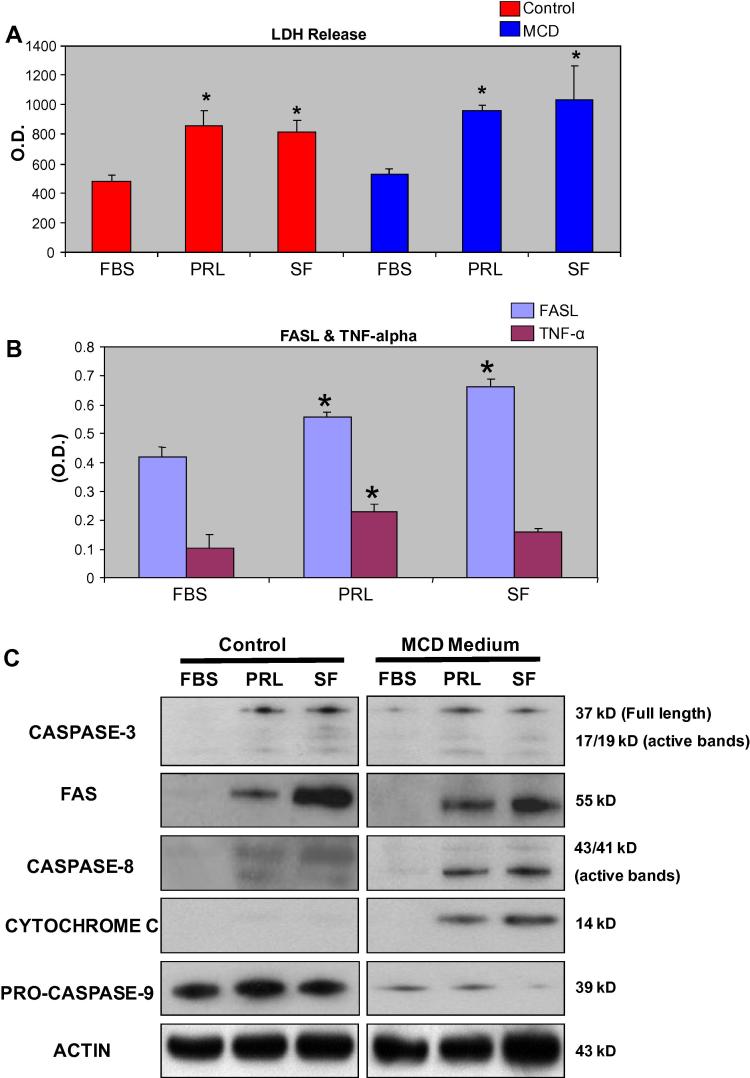
(A, B, C): PRL induces hepatocytes injury in vitro. Mouse primary hepatocytes were cultured for 12 h in control media, MCD deficient or serum free (SF) media to simulate NASH in vitro, in the presence or absence of PRL. At completion LDH was analyzed. As shown (A) PRL treatment induces hepatocyte injury, reflected by release of LDH in both control and or MCD media. As confirmation of induction of death inducing pathways we also assayed FASL and TNF-α after hepatocyte culture in control medium. As shown in (B), PRL induced release of the pro-apoptotic proteins FASL and TNF-α from cultured primary hepatocytes. Analysis of the apoptotic pathway proteins in the presence of PRL showed that in MCD media, PRL drives hepatocyte apoptosis via the upregulation of FAS, caspase-8 and cytochrome c (C).

LDH release was significantly increased by PRL or serum free (SF) media with control or MCD media (Fig. 3A).

### 3.4 PRL induces release of FASL and TNF-α from hepatocytes

Since PRL increased hepatocyte death, we next sought to determine whether the pro-apoptotic proteins TNF-α and FASL were released into the culture medium in the presence of PRL. Both proteins act as ligands for the initiation of the apoptotic pathway by interacting with the death receptor, FAS [21], [22]. We show here a robust release of FASL from hepatocytes cultured with PRL (10 μM) (Fig. 3B). Release of TNF-α, assayed as an alternative pro-apoptotic protein, was similarly enhanced by PRL (Fig. 3B).

### 3.5 PRL treatment induces hepatocyte apoptosis via the apoptotic pathways

Since PRL induced death ligands release as shown above, we next studied involved death pathways. Primary murine hepatocytes were again cultured in control or MCD medium with or without PRL. Cell lysates were then analyzed for changes in expression of proteins involved in the apoptotic pathway.

The apoptotic effector protein caspase-3 was activated in both the PRL and SF treated cells, in control or MCD media, indicating that an apoptotic program of death is induced by treatment with PRL (Fig. 3C). PRL treated groups displayed prominent levels of the apoptotic pathway death receptor Fas (Fig. 3C). Similarly, death receptor dependent protein caspase-8 was activated, as judged by the appearance of its cleavage products (43/41 kDa). The effect was observed equally in control and MCD media.

Cytochrome c was not readily detected after hepatocyte culture in control medium, but considerable levels were observed in MCD media after PRL treatment or under SF conditions (Fig. 3C). Similarly, the reduction of caspase-9 from its inactive pro-form to active form was only observed in MCD media (Fig. 3C). Thus, PRL promotes hepatocyte death via the apoptotic pathway under conditions, which more closely mimic the in vivo situation in NASH.

## 4 Discussion

Currently, the most prevalent chronic liver disease in affluent countries is NAFLD but despite its clear demographic importance, there are no proven treatments, reflecting lack of understanding of the mechanisms underlying the disease.

Our previous studies have shown that the α1-adrenoceptor antagonist, PRZ, reduced liver injury in a mouse model of NASH [7]. SNS signaling appears to also be regulating hepatic fibrogenesis through effects on hepatic fibrogenic cells [8], [9], [23], which offers the prospect that adrenoceptor antagonists may be used as pro-regenerative, anti-fibrotic agents in liver disease. This prospect is alluring because there is extensive clinical experience in liver disease, with β-adrenoceptor antagonists, such as PRL, which are widely used as treatments for portal hypertension [11], [12]. Our hypothesis here was that PRL would as with PRZ reduce liver injury. If our hypothesis was proven, SNS antagonism with PRL as a novel, early, pro-regenerative treatment for NAFLD and potentially other liver diseases could easily be translated to the clinic because of the vast experience with this and other β-adrenoceptor antagonists and their low cost compared to putative cytokine or cell based therapies.

We tested our hypothesis by first studying the effect of the β-adrenoceptor antagonist, PRL in a murine model of NASH. Biochemical evidence of liver injury was determined by assaying ALT release and confirmed by histological evaluation for necrosis and steatosis. Our results show that PRL, unlike PRZ, increased biochemical and histological markers of liver injury and cell death, under conditions simulating NASH.

To ascertain whether PRL induced hepatocyte death and to analyze the mechanisms therein, primary hepatocytes were first confirmed to express adrenoceptors. Hepatocytes were then cultured in a medium deficient in methionine and choline, to simulate in vitro, the in vivo conditions of increased oxidant stress that occur with NASH, which replicates the HMCDE murine model of NASH. These studies confirmed that PRL, in vitro as well, induced hepatocyte death as evidenced by increased release of LDH, TNF-α and FAS ligand. To then determine the pathways induced by PRL, we studied factors from the apoptotic pathway. In control media, PRL induced death predominantly via the upregulation of Fas receptor and caspase-8 proteins, whereas in MCD deficient media, PRL similarly induced death through upregulation of FAS, caspase-8 as well as cytochrome c, which was not upregulated in the control group.

Therefore our starting hypothesis that PRL similar to PRZ would reduce liver injury was not correct. PRL increased liver injury in mice fed NC but more so in animals fed a NASH simulating HMCDE diet through activating the apoptotic pathway in hepatocytes. Moreover, the β-AR antagonist, PRL, whilst useful for portal hypertension may be detrimental to the liver in the absence of portal hypertension, in NASH. As such, α-adrenoceptor but not β-AR antagonists offer novel clinical utility as pro-regenerative, anti-fibrotic therapeutic agents in NAFLD. These findings are clinically significant in the increasing population of patients with metabolic syndrome and obesity, who have ischemic heart disease and are placed on β-blockers but may concurrently have undeclared NASH. Further studies in animals and humans are required to study the effects of β-blockers in the context of NAFLD and NASH. Meanwhile, β-AR antagonists should be avoided or used with caution in individuals with NASH to prevent worsening liver injury.
